# Domestic violence against women in Jordan: analysis of the demographic and health survey dataset 2017-2018

**DOI:** 10.25122/jml-2023-0111

**Published:** 2023-08

**Authors:** Khalid Ahmed Kheirallah, Ahmad Alrawashdeh, Akram Alsaleh, Mahmoud Megdadi, Sara Obeidat, Khaled Abdulraheem Elfauri, Abdel-Hameed Al-Mistarehi, Iffat Elbarazi

**Affiliations:** 1 Department of Public Health and Family Medicine, Faculty of Medicine, Jordan University of Science and Technology, Irbid, Jordan; 2 Department of Allied Medical Sciences, Faculty of Applied Medical Sciences, Jordan University of Science and Technology, Irbid, Jordan; 3 Faculty of Medicine, Jordan University of Science and Technology, Irbid, Jordan; 4 Johns Hopkins University School of Medicine, Baltimore, Maryland, USA; 5 Institute of Public Health, College of Medicine and Health Sciences, United Arab Emirates University, Al Ain, Abu Dhabi, United Arab Emirates (UAE)

**Keywords:** domestic violence, DHS, women, Jordan, GBV, IPV, wealth index, socio-economic status, WHO: World Health Organization, DV: Domestic Violence, DHS: Demographic and Health Survey, EMR: Eastern Mediterranean Region, CTS: Conflict Tactics Scale, PCA: Principal Component Analysis, SES: Socio-Economic Status, AOR: Adjusted odds ratio, VIF: Variance Inflation Factor, ICF: International Classification of Functioning, Disability, and Health, STROBE: Strengthening the Reporting of Observational Studies in Epidemiology

## Abstract

This study analyzed the 2017-2018 Jordan Demographic and Health Survey (DHS) database to determine the prevalence of domestic violence (DV) against women in Jordan and its associated sociodemographic factors. The findings revealed that among Jordanian women, the lifetime prevalence of DV by husbands was 25.9%, with emotional (20.6%), physical (17.5%), and sexual (5.1%) violence being prominently reported. DV against women was significantly associated with the age, region, and educational status of women, as well as the wealth index, but not their husbands. While the results suggest a potential reduction in DV estimates compared to the last decade, DV still represents a public health issue in Jordan. The study highlights the direct association of DV with socio-demographic characteristics and provides a gateway to identifying high-risk women and implementing appropriate interventions to reduce DV.

## INTRODUCTION

The World Health Organization (WHO) defines violence as “the intentional use of physical force or power, threatened or actual, against oneself, another person, a group, or society that either result in or has a high probability of resulting in injury, death, psychological damage, maldevelopment or deprivation” [1]. Violence against women is a widespread issue that significantly contributes to their ill health. Domestic violence (DV) is the most common form of violence against women, which is usually committed by someone in the victim's domestic circle [2].

DV affects the social, sexual, and reproductive health aspects of millions of women and families [1, 3]. DV is widely acknowledged as a serious human rights violation and a growing global public health problem. It is estimated that between 20% and 50% of women witness DV globally, with estimates ranging from 15%, in Japan, to 71% in rural Ethiopia [2-4]. DV results from unequal power relationships between men and women and is typically perpetrated by the husband/intimate partner in the domestic sphere. While DV is a global phenomenon, women living in poverty or between the ages of 16 and 24 are more likely to experience such events [3, 4].

Jordan, a developing country in the WHO’s Eastern Mediterranean Region (EMR), has limited updated studies that address the prevalence of DV against women, and little is known about the magnitude of this problem and its burden and patterns at the national level. Few studies were conducted at a regional scale [5, 6] or within healthcare settings in 2010 [7-9]. Previous data collected during the last decade indicated that intimate partner violence and emotional abuse (e.g., shouting and insulting) were common in some governorates (87% and 47.5%, respectively). Wife beating, reported at 19.6%, was indicated by women as being “justified” as “a way of disciplining women” [5]. In a recent systematic review and meta-analysis analyzing a large sample of 19,101 reproductive health attendee women from 10 Arab countries, the prevalence lifetime estimates of women who have undergone at least one type of the following intimate partner acts of violence were: 97.2% control violence, 73.4% psychological violence, 31.2% physical violence, and 18.8% sexual violence [8]. DV prevalence estimates among women utilizing maternal and child health services in urban settings were 39%, 30%, and 6% for emotional, physical, and sexual abuse patterns, respectively [9]. The prevalence rates of violence by the partner during pregnancy, including verbal, physical, emotional, and sexual violence, were estimated at 10.4%, 23.4%, 23.7%, and 5.7%, respectively [6]. Still, women in this study felt “vulnerable, embarrassed, afraid, prisoner, and stigmatized” after each abusive incident [6].

Official reports may be available, but their reflection on the problem is limited to counting incidents of domestic violence (DV) when they result in severe injury or death. While Jordan has been witnessing a rapid increase in the number of cases of violence against women, recent reports indicated that official DV estimates are “not enough” to curb such behaviors and highlighted the “outburst of violence against Jordanian women, where the country recorded 21 cases of female murders – a three-time increase compared to 2018 which saw seven murders only” [10].

The conservative nature of the societal attitudes towards gender roles within the culture and the fact that such roles heavily depend on “the basics of social structure” are critically important when dealing with DV in Jordan. For example, DV may still be considered “a personal and familial issue” rather than a “social and legal problem”. Dissemination of family violence, including DV, information within the local community, friendship zones, and extended families, is expected to stigmatize family members and damage family reputation, unity, and dignity. This creates reporting issues as women tend not to report such incidents officially and, most importantly, not seek proper assistance outside their household, as this will be considered taboo. Keeping DV issues internal not only reduces the number of reported incidents of violence against women but also creates a culture that “blames, mainly, the victim and, to a lesser tendency, blames the abuse on the husband, marital problems, as well as familial and societal conditions”. Reports from Jordan have highlighted the alarming victimization of Jordanian women, encompassing physical, psychological, and sexual abuse. These abusive behaviors are found across diverse cultural and social settings, including within families, universities, and workplaces [5]. Thus, this study aimed to estimate the national prevalence of DV in Jordan and assess the social patterning of such behavior(s) to identify proper channels for culturally tailored interventions considering that most studies in literature dealing with this issue are from Western cultures.

## MATERIAL AND METHODS

This is a descriptive, cross-sectional, analytical study of the 2017-2018 Jordan Demographic and Health Survey (DHS) dataset. This survey is an internationally recognized program that uses nationally representative questionnaires from over 90 developing countries. The survey primarily targets ever-married women of reproductive age, between 15 and 49 years old, and covers various aspects of their lives, such as background characteristics, reproductive behavior, perinatal care, children's health, and women's empowerment. In Jordan, the 2017 dataset included 14,689 ever-married women. From this dataset, a sub-sample of ever-married women was specifically interviewed to gather information related to the domestic violence (DV) module. To ensure national representation, one woman was randomly selected from each eligible household, and the data were weighted using designed weights provided within the dataset. With this constraint, a weighted sample of 6,852 ever-married women in Jordan was used for the current analysis.

### Measurement of DV in Jordan’s DHS data module

Spousal/partner violence, using a modified Conflict Tactics Scale (CTS) approach [11], was assessed in greater detail than violence by other perpetrators. This approach involves implementing a modified version of the original CTS [12] to capture more information on spousal/partner violence using a series of questions to reach the violence experienced. The modified list used by DHS includes around 15 acts, defined as “a series of individual questions regarding specific acts of violence” and categorized under physical, emotional, and sexual violence. If the respondent affirms that any specified acts or outcomes have occurred, she is considered to have experienced violence [11]. In particular, the spousal violence of the husband/partner for currently married women, and the most recent husband for formerly married women, was evaluated by posing a series of questions (Table 1) that intend to assess domestic violence represented by physical, emotional, and sexual domains.

**Table 1:** Domestic violence domains and questions used in the Demographic and Health Survey (DHS) dataset

Domain	Questions: Does (did) your (last) husband/partner ever:
Emotional violence	a) Say or do something to humiliate you in front of others? b) Threaten to hurt or harm you or someone close to you? c) Insult you or make you feel bad about yourself?
Physical violence	d) Push you, shake you, or throw something at you? e) Slap you? f) Twist your arm or pull your hair? g) Punch you with his fist or something that could hurt you? h) Kick you, drag you, or beat you up? i) Try to choke you or burn you on purpose? j) Threaten or attack you with a knife, gun, or another weapon?
Sexual violence	k) Force you to perform any sexual acts you did not want to?

A “yes” answer to one or more of the items in each of the DV domains constitutes evidence of violence within that specific domain, namely “lifetime experience of violence” [12]. Additionally, the data differentiates between "less severe violence" when there is a "yes" response to one or more of items "d" to "g" and "severe violence" when there is a "yes" response to one or more of items "h" to "j" [13].

Exposure to any item in each DV domain was considered positive for violence. The outcome variables, emotional, physical, and sexual violence, were all treated as binary variables. Those who reported experiencing violence were coded as 1, while those who reported never experiencing violence were coded as 0 for each DV domain. Furthermore, the presence of physical violence, emotional violence, and sexual violence constituted DV.

### Social patterning

The main socio-demographic attributes considered were abstracted from available DHS data files, including the area of residence (urban-rural), region (north-middle-south), age group, women’s educational level, wealth quintiles, and husband’s educational status. The wealth index, also known as the DHS wealth index, is constructed based on household ownership of assets, goods, and services using the Principal Component Analysis (PCA) statistical technique [14]. The index is considered a valid measure of Socio-Economic Status (SES) based on a list of household characteristics [15]. It has also been used in epidemiological studies and national and sub-national surveys to measure SES, leading to a significant and welcome increase in the analysis of inequalities [15]. A recent review concluded that wealth indices based on household assets are “valid but distinct” from income and consumption measures. Further, it was indicated that the wealth index is based on assets acquired over time rather than on income/expenditure at one point in time. This approach may better relate to behavior patterns established over the years, which household surveys often try to measure [16].

### Public involvement

Original DHS data involved community leaders and public members for questionnaire design, data collection activities, and dissemination of results. The current study research questions were openly discussed with public health experts, community leaders, and non-state actors to review the data analysis plan, results, discussion points of view, and dissemination of results.

### Statistical analysis

Country-specific DHS datasets are publicly available from www.measuredhs.com. Data was first downloaded for Jordan before eligible participants were selected, and a dataset appropriate for analysis was created. DV weight variable was then applied, and frequency analysis was presented using numbers and percentages for each variable. Cronbach’s alpha was also reported to assess each domain’s reliability. DV domains assessed were emotional, physical, and sexual violence. Accordingly, every exposure to DV was determined for each domain of DV. Prevalence estimates of each DV domain were reported. A Chi-square test was used to assess the potential relationships between social patterning variables and exposure to each DV domain. Multivariate logistic regression models were used to assess the association of each DV domain, including emotional, physical, and sexual violence, with different socio-demographic patterning variables, including age, education, husband/partner education, household wealth, region, and residential area. The backward stepwise (conditional) selection was used for each model. Adjusted odds ratio (AOR) and 95% confidence intervals (95% CI) were reported. Multicollinearity was assessed using a Variance Inflation Factor (VIF) for independent variables (none above 5). A p-value of less than or equal to 0.05 was considered statistically significant. All analyses were performed using SPSS version 21 software.

### Ethical Considerations

The survey procedures and instruments used for the study were ethically approved by the ethics committee of ICF Macro International, Inc, Calverton, Maryland, USA, and by the National Ethics Committee of each country. To conduct this study, permission was received from the registered DHS Program-Data Archive website at the International Classification of Functioning, Disability, and Health (ICF). The study was conducted in accordance with the Declaration of Helsinki of 1975, as revised in 2008, and the WHO guidelines for interviewing women for DV were also followed [17]. Informed verbal consent was obtained from each participant before the interview. This work has been reported based on STROBE (Strengthening the Reporting of Observational Studies in Epidemiology) guidelines [18].

## RESULTS

A total of 6,852 ever-married women were included in the current analysis, of which more than half (53.8%) were between 25 and 39 years old, the majority (90.1%) were living in urban areas, and about two-thirds (62.5%) were living in the central region of Jordan. Distribution by education showed almost equal distribution among the three educational attainment groups: less than high school diploma (Tawjehi), high school diploma, and post-high school diploma (38.3%, 26.8%, and 34.9%, respectively). More than two-thirds of women had a partner with secondary education (53.5%) or less (11.6%). Almost equal distribution was observed for the wealth index (Table 2).

**Table 2:** Frequency distribution of socio-demographic characteristics of respondents

Study Variable	Number (n=6,852)	%
**Age (years)**		
15-24	903	13.2
25-39	3,687	53.8
40-49	2,262	33.0
**Type of place of residence**		
Urban	6,175	90.1
Rural	677	9.9
**Region**		
Central	4,283	62.5
North	1,916	28.0
South	653	9.5
**Highest educational level**		
Less than Tawjehi*	2,625	38.3
Tawjehi	1,837	26.8
More than Tawjehi	2,390	34.9
**Husband/partner’s education level**		
Primary and less	793	11.6
Secondary	3,654	53.3
Higher than Secondary	1,946	28.4
Total	6,393	93.3
Missing	459	6.7
**Wealth index**		
Poorest	1,336	19.5
Poorer	1,424	20.8
Middle	1,430	20.9
Richer	1,495	21.8
Richest	1,168	17.0

* Tawjehi is equivalent to high school

Cronbach’s alpha for physical and emotional violence items was 0.807 (n=7 items) and 0.723 (n=3 items), respectively. The distribution of DHS DV items is presented in Table 3. Two out of seven physical violence items had a prevalence estimate (yes *vs*. no) of more than 10%; “Ever been pushed, shook or had something thrown” (11.6%) and “Ever been slapped by husband/partner” (11.2%). Both “Ever had arm-twisted or hair pulled” and “Ever been punched with a fist or hit by something harmful” were reported by around 6% for each. As defined by the DHS, physical violence severity was reported at 4.3% for “severe”, compared with 17.0% for “less severe” physical violence. While 82.5% of women reported not experiencing any item of physical violence, 7.1% and 4.4% of participants reported ever having only one and two items, respectively, of such violence, while 0.3% reported ever having all seven items of physical violence.

**Table 3:** Frequency of domestic violence items in Jordan’s Demographic and Health Survey (DHS) dataset

Item	Number (%)				
	Often	Sometimes	Yes, but not in 12 months	Never	Total
**Physical violence Domain**					
**Pushed, shook, or had something thrown by husband/partner**					
	161	424	213	6,055	6,853
	2.3%	6.2%	3.1%	88.4%	100.0%
**Slapped by husband/partner**					
	123	363	280	6,085	6,851
	1.8%	5.3%	4.1%	88.8%	100.0%
**Arm twisted or hair pulled by husband/partner**					
	100	166	154	6,432	6,852
	1.5%	2.4%	2.2%	93.9%	100.0%
**Punched with a fist or hit by something harmful by husband/partner**					
	84	188	139	6,441	6,852
	1.2%	2.7%	2.0%	94.0%	100.0%
**Kicked or dragged by husband/partner**					
	70	107	71	6,604	6,852
	1.0%	1.6%	1.0%	96.4%	100.0%
**Strangled or burnt by husband/partner**					
	30	45	28	6749	0
	0.4%	0.7%	0.4%	98.5%	0.0%
**Threatened with a knife, gun, or another weapon by husband/partner**					
	24	28	14	6786	6852
	0.4%	0.4%	0.2%	99.1%	100.0%
**Emotional Violence Domain**					
**Humiliated by husband/partner**					
	204	496	221	5931	6852
	3.0%	7.2%	3.2%	86.6%	100.0%
**Threatened with harm by husband/partner**					
	109	176	88	6479	6852
	1.6%	2.6%	1.3%	94.6%	100.0%
**Insulted or made to feel bad by husband/partner**					
	195	642	258	5757	6852
	2.8%	9.4%	3.8%	84.0%	100.0%
**Sexual Violence Domain**					
**Physically forced into unwanted sex by husband/partner**					
	60	169	120	6503	6852
	0.9%	2.5%	1.8%	94.9%	100.0%

In terms of emotional violence, the item with the highest prevalence estimate was “Ever been insulted or made to feel bad” (16.0%), followed by “Ever been humiliated” (13.4%) and “Ever been threatened with harm” (5.4%). While 79.4% of women reported not experiencing any form of emotional violence, 9.7% reported experiencing only one item, and 7.3% reported two items of such violence. Ever experiencing any emotional item violence was estimated at 20.6%. All three items were experienced by 3.5% of participants. Sexual violence item was estimated at 5.1% (Table 3).

Overall, about three-quarters of women did not experience any of the above items. The prevalence of women who reported experiencing any DV (emotional, physical, or sexual) was 25.9%. The prevalence of participants experiencing emotional violence (any item) was 20%, while 17.5% experienced physical violence (any item). Physical-only, emotional-only, and sexual-only prevalence estimates were 3.9%, 7.0%, and 0.9%, respectively. While 3.2% of participants reported experiencing all forms of violence, 9.9% reported combined physical and emotional violence.

The distribution of study participants by background characteristics and DV domains is presented in Table 4. Emotional and sexual, but not physical, violence increased with the higher age group (p=0.026 and p=0.028, respectively). Women living in urban areas seem to have significantly higher estimates of physical violence (p=0.021) than those living in rural areas. Women residing in the Central and Northern parts of Jordan also seem to have higher levels of all types of violence (p<0.001 for all comparisons). Women with lower education were found to have higher levels of all types of violence (p<0.001 for all comparisons). The higher educational status of partners was associated with lower estimates of all types of domestic violence (p<0.001 for all comparisons). Significant differences in violence estimates were also detected by the wealth index.

**Table 4:** Differences in domestic violence domains by social patterning of participants

	DV Domain													
	Emotional violence				Physical violence				Sexual violence					
	No		Yes		No		Yes		No		Yes		Total	
	N	%	N	%	N	%	N	%	N	%	N	%	N	%
**Age (years)**														
15-24	738	81.7	165	18.3	765	84.8	137	15.2	872	96.7	30	3.3	903	100.0
25-39	2,947	79.9	741	20.1	3,012	81.7	675	18.3	3,484	94.5	203	5.5	3,688	100.0
40-49	1,759	77.8	503	22.2	1,874	82.8	388	17.2	2,146	94.9	116	5.1	2,262	100.0
Total	5,444	79.4	1,409	20.6	5,651	82.5	1,200	17.5	6,502	94.9	349	5.1	6,853	100.0
p-value	0.026				0.075				0.028					
**Type of place of residence**														
Urban	4,894	79.3	1,281	20.7	5,071	82.1	1104	17.9	5,861	94.9	314	5.1	6,175	100.0
Rural	550	81.2	127	18.8	580	85.7	97	14.3	642	94.7	36	5.3	677	100.0
Total	5,444	79.5	1,408	20.5	5,651	82.5	1,201	17.5	6,503	94.9	350	5.1	6,852	100.0
p-value	0.225				0.021				0.801					
**Region**														
Central	3,274	76.4	1,010	23.6	3,395	79.3	888	20.7	4,028	94.0	255	6.0	4,283	100.0
North	1,602	83.6	314	16.4	1,669	87.1	247	12.9	1831	95.6	85	4.4	1,916	100.0
South	568	87.0	85	13.0	587	89.9	66	10.1	643	98.5	10	1.5	653	100.0
Total	5,444	79.4	1,409	20.6	5,651	82.5	1,201	17.5	6,502	94.9	350	5.1	6,852	100.0
p-value	<0.001				<0.001				<0.001					
**Woman’s highest educational level**														
Less than Tawjehi	1,946	74.2	678	25.8	2031	77.4	594	22.6	2471	94.2	153	5.8	2,624	100.0
Tawjehi	1,462	79.6	375	20.4	1,520	82.7	317	17.4	1,735	94.4	102	5.6	1,837	100.0
More than Tawjehi	2,035	85.1	355	14.9	2,100	87.8	290	12.2	2,297	96.1	94	3.9	2,391	100.0
Total	5,444	79.5	1,407	20.5	5,651	82.5	1,201	17.5	6,503	94.9	350	5.1	6,852	100.0
p-value	<0.001				<0.001				0.005					
**Husband/partner’s highest education level**														
Primary or less	581	73.3	212	26.7	602	76.0	190	24.0	764	94.1	47	6.4	792	100.0
Secondary	2,901	79.4	753	20.6	3,012	82.4	643	17.6	3478	95.2	176	5.6	3,655	100.0
Higher than Secondary	1,699	87.3	247	12.7	1,772	91.1	174	8.9	1,882	96.7	64	4.8	1,946	100.0
Total	5,181	81.0	1,212	19.0	5,386	84.2	1,007	15.8	6,106	95.5	287	4.5	6,393	100.0
p-value	<0.001				<0.001				0.004					
**Wealth index**														
Poorest	1,007	75.4	328	24.6	1,047	78.4	289	21.6	1,246	93.3	90	6.7	1,336	100.0
Poorer	1,087	76.3	337	23.7	1,150	80.8	273	19.2	1,354	95.2	69	4.8	1,423	100.0
Middle	1,176	82.3	253	17.7	1,222	85.5	207	14.5	1,367	95.6	63	4.4	1,430	100.0
Richer	1,198	80.1	298	19.9	1,250	83.6	245	16.4	1,426	95.4	69	4.6	1,495	100.0
Richest	976	83.6	192	16.4	982	84.1	186	15.9	1,109	95.0	58	5.0	1,167	100.0
Total	5,444	79.5	1,408	20.5	5,651	82.5	1,200	17.5	6502	94.9	349	5.1	6,851	100.0
p-value	<0.001				<0.001				0.044					

The adjusted effects of social patterning attributes for each DV domain are presented in Table 5. The odds of emotional violence increased with increasing age groups. Compared to residents in the central region of Jordan, those in the northern and southern parts reported lower odds of emotional violence. Women who reported having more than secondary (Tawjehi) and secondary education were significantly less likely to experience emotional violence than those in the less-than-high school educational category. Women in the middle, richer, and richest wealth index groups were significantly less likely to experience emotional violence.

**Table 5:** Adjusted effects of socio-demographic and economic characteristics on emotional, physical, and sexual violence

	DV Domain					
	Emotional		Physical		Sexual	
	AOR	95% CI	AOR	95% CI	AOR	95% CI
**Age (years)**						
15-24	1.00	Ref.	1.00	Ref.	1.000	Ref.
25-39	1.33	(1.10 - 1.62)	1.49	(1.21 - 1.83)	1.91	(1.28 - 2.83)
40-49	1.36	(1.11 - 1.67)	1.15	(0.92 - 1.44)	1.68	(1.10 - 2.55)
**Region**						
Central	1.00	Ref.	1.000	Ref.	1.000	Ref.
North	0.56	(0.49 - 0.65)	0.51	(0.43 - 0.60)	0.68	(0.52 - 0.88)
South	0.46	(0.36 - 0.58)	0.42	(0.32 - 0.55)	0.23	(0.13 - 0.45)
**Highest educational level (women)**						
Less than Tawjehi	1.00	Ref.	1.00	Ref.	1.00	Ref.
Tawjehi	0.78	(0.67 - 0.91)	0.70	(0.60 - 0.83)	1.00	(0.76 - 1.32)
More than Tawjehi	0.55	(0.47 - 0.65)	0.47	(0.39 - 0.55)	0.68	(0.51 - 0.91)
**Wealth index**						
Poorest	1.00	Ref.	1.00	Ref.	1.00	Ref.
Poorer	0.98	(0.82 - 1.09)	0.90	(0.75 - 1.09)	0.71	(0.51 - 0.98)
Middle	0.68	(0.57 - 0.83)	0.65	(0.53 - 0.80)	0.63	(0.45 - 0.89)
Richer	0.76	(0.63 - 0.92)	0.74	(0.60 - 0.90)	0.64	(0.45 - 0.89)
Richest	0.59	(0.47 - 0.73)	0.72	(0.57 - 0.89)	0.69	(0.47 - 0.99)

AOR: Adjusted Odds Ratio; 95% CI: 95% Confidence Interval; Ref: Reference category.

For physical violence, women aged 25 to 30 years were significantly more likely to have been exposed to physical violence than those in the 15 to 24 age group. Residents in the northern and southern parts of Jordan were significantly less likely to experience domestic violence than those in the central part. Higher educational levels and higher wealth indexes were significantly associated with lower odds of physical violence. Similar findings were also found for sexual violence.

## DISCUSSION

This study is the first to explore the prevalence estimates of DV, namely physical, emotional, and sexual violence items, and investigate the social patterning of selected variables, including wealth index and educational levels of women and their partners, utilizing a national sample of ever-married women from the 2017-2018 Jordan DHS dataset. The prevalence of ever-married women who reported experiencing any DV item, including emotional, physical, or sexual violence, was 25.9%. One in every four ever-married women in Jordan reported being exposed to DV. The most common type of DV experienced by women was emotional, followed by physical violence. One in every six women reported ever being exposed to physical violence (17.5%), one in every five women in Jordan reported ever being exposed to emotional violence (20.6%), and one in every 20 women reported ever being exposed to sexual violence (5.1%). DV was inversely associated with higher social class, measured by wealth index, and higher educational levels among women. The partner’s educational level was not a significant predictor of any type of DV.

Within the Arab countries, one systematic review pointed out the need to present more data about domestic violence and described available prevalence estimates as “fragmented” and “out-of-date”. DV rates were reported to vary widely across surveys but were in line with the WHO estimate of 37% for physical and/or sexual against ever-partnered women in WHO’s Eastern Mediterranean region [19]. While these estimates may be higher than that reported in the current study, they all reflect a substantial public health problem that needs a regional research agenda to monitor change over time and better understand the magnitude of violence against women. On the other hand, variability in the reported estimates reflects the diversity of tools and definitions used to assess domestic violence. Thus, caution should be considered for comparison.

The “social culture” still accepts violence against women as a kind of “discipline” in many countries worldwide. This problem has social and health ramifications affecting different societies and cultural and ethnic groups. In Jordan, while this may be acceptable at the cultural and social levels, it is not acceptable from a religious point of view. Accordingly, research on domestic violence is a multi-dimensional problem. Future research may better reflect on this problem by utilizing religious attributes and exploring potential pathways that perpetuate violence. Building a stronger case against DV should invest in effective policies and programs that raise awareness and engage men and women using an evidence-based approach such as that provided in the current study.

DV estimates usually mirror unequal power between men and women [20], and the “persistent” excuse of DV by women, reported in developing countries, suggested that many women who live under “classic patriarchy” usually conform to the “norms of wife blaming” as a coping mechanism against DV [21, 22]. Women also tend to internalize the idea that a husband who physically punishes or verbally reprimands his wife is exercising his “rights”. Such rights are also perceived as “in a woman’s interest”, and a legitimate reprisal for a wife’s disobedience rather than violence [21, 22]. Hence, in such societies, the justification of DV might be used to understand the high DV estimates and the disproportionate estimates between developed and developing countries. Empowering women and improving their participation in socio-political life could be considered suitable means to reduce DV via changing attitudes and providing balanced relationships between males and females [23].

In Jordan, gender perceptions are changing, and the traditional gender role may be fading. This may explain the lower DV estimates reported in the current study compared to those reported around 2010. Still, our results are based on a national sample and were not restricted to a sub-regional assessment or women attending healthcare settings. Moreover, the time difference between our study and previous ones, spanning a decade, could also account for the variations in DV estimates. During the last decade, advancement in the civil rights movements in Jordan may have been a reason for the lower estimates reported in our study compared to previous reports from Jordan [5, 8, 9]. Jordan has witnessed an upsurge of women’s rights movements and non-state actors-initiated programs to increase awareness of women’s rights and raise their voices and participation.

This study suggested that urban-rural differences were insignificant predictive factors for domestic violence as urban women did not have a different chance of experiencing violence than rural women. This finding is consistent with a cross-sectional household survey conducted by Andersson *et al*. in eight southern African countries, which showed no significant difference in DV between women living in rural and urban settings [24]. Still, the detected regional differences in violence against women in the current study are crucial, as women living in the southern and northern parts of Jordan were at lower risk of violence than those living in the central part. Such geographic differences are critical in designing interventions to reduce DV and were reported elsewhere [25].

The study revealed a significant decrease in the likelihood of experiencing domestic violence with an increase in the educational status of women. This is consistent with previous findings [26, 27] and contradicts other reports [28, 29]. Maternal education may be seen here as a protective factor, as educated women are better equipped not only to challenge the classical social norms around gender roles and expectations of women and men but also to contest the justification for DV. Additionally, educated women may have greater financial independence, which can help alleviate tensions between “traditional” and “modern” gender roles. Financial dependence has been suggested as a factor that can normalize domestic violence [23].

Previous reports suggested that discriminatory formal and informal social institutions [23], including gender norms and the acceptance of DV, are deeply rooted and cannot be separated from their geographical, socio-cultural, economic, and political settings. Female socio-economic empowerment was also suggested as a protective factor against DV [23]. In the current study, the wealth index was inversely associated with DV, indicating that economically poor households are at a higher risk of DV. This finding is consistent with other reports where deprivation and low family income were associated with a higher risk of violence and contributed significantly to developing emotional and behavioral problems [29, 30]. Jordan suffers from limited natural resources, high unemployment rates, job competition, and economic challenges worsened by the Syrian and Iraqi crises [30]. Food insecurity and childhood traumas were reported to shape pathways to substance misuse and poor mental health, increasing DV, whereas higher education could increase gender-equitable attitudes and reduce DV [31]. However, evidence from DHS surveys did not support a clear relationship between women's asset ownership and experience of DV. While asset ownership was negatively associated with DV in three countries, it was positively associated and had no significant relationship in five and 20 countries, respectively. Regardless, our finding suggests that poor households may be a target for DV programs and interventions. This was also reported elsewhere [32].

The results of the multivariate regression models showed that the husband's characteristics were not significantly associated with DV domains, despite showing significance in the univariate analyses. In this regard, the literature is inconsistent as the role of the husband's characteristics was inconclusive with DV. While the risk of DV was higher among less-educated women and their partners in 10 out of 14 sites in a multi-country investigation [1], neither women’s nor husbands’ educational levels were associated with DV among pregnant women in Jordan in 2008 [7]. Indeed, educated women tend to marry more highly educated men. If this is true, the current results may reflect a more potent effect of women's education than their partner’s education. The fact that the partner’s education was not a significant predictor of DV in Jordan further strengthens the effect of educating women as a tool to empower them and reduce the risk of DV.

Younger women may be more vulnerable to DV than older women [33]. Still, findings in this regard are not consistent. While some studies reported that adolescent girls were at higher risk of DV [34, 35], others indicated that older women were at higher risk [24, 25, 33]. In our current analysis, we found that older women were at a higher risk of experiencing DV compared to younger women. This finding is interesting and needs to be understood within the cultural context. While young women have a better understanding of the criminal nature of DV than older women do, they are less likely to understand its complexities in relationships of range and seriousness. As such, younger women may report more incidents of violence, while older women may not. Such information bias would have increased the DV estimates for younger women and decreased estimates for older ones.

### Limitations of the study

This study is limited by the nature of the secondary data collected in the DHS. However, this study used a standardized tool to assess the dimensions of domestic violence, making the results comparable to other studies that used secondary data. Another limitation of this study is the self-reporting of a socially undesirable issue that may be associated with cultural taboo and sensitivity towards it. As a result, under-reporting of domestic violence items is expected. This under-reporting could vary based on cultural and social norms and participants' characteristics, such as age. However, the conduct of this study was through trained staff, and the interview was completed in a private place to ensure the confidentiality of responses. The comparison between the current study and other original studies may be invalid. However, different assessment measures and dimensions of social patterning attributes may affect the study findings, especially in the regression model.

## CONCLUSION

The literature showed mixed relationships between DV and its correlates. The country-specific particularities should then be carefully considered for policy programming at the national level. Our findings provide evidence of a reduction in domestic violence among women in Jordan during the last decade and show potential social attributes that could be of interest when designing future research and intervention measures. Women’s vulnerability to DV seems to increase with decreasing wealth index and educational status.

## Data Availability

All data generated and analyzed during this study are publicly available and can be provided upon request from the corresponding author.

## Personal thanks

The authors would like to extend their appreciation to those who made the DHS datasets possible.

## References

[1] Abramsky T, Watts CH, Garcia-Moreno C, Devries K (2011). What factors are associated with recent intimate partner violence? findings from the WHO multi-country study on women's health and domestic violence. BMC Public Health.

[2] World Health Assembly (2014). Addressing the global challenge of violence, in particular against women and girls, and against children: Report by the Secretariat. 67th World Heal Assem.

[3] Semahegn A, Mengistie B (2015). Domestic violence against women and associated factors in Ethiopia; systematic review. Reprod Health.

[4] Naidoo K, Adeagbo O, Pleaner M (2019). Sexual and reproductive health needs of adolescent girls and young women in sub-Saharan Africa: Research, policy, and practice. SAGE Open.

[5] Al-Nsour M, Khawaja M, Al-Kayyali G (2009). Domestic violence against women in Jordan: Evidence from health clinics. J Fam Violence.

[6] Oweis A, Gharaibeh M, Alhourani R (2010). Prevalence of violence during pregnancy: findings from a Jordanian survey. Matern Child Health J.

[7] Clark CJ, Silverman J, Khalaf IA, Ra'ad BA (2008). Intimate partner violence and interference with women's efforts to avoid pregnancy in Jordan. Stud Fam Plann.

[8] Clark CJ, Bloom DE, Hill AG, Silverman JG (2009). Prevalence estimate of intimate partner violence in Jordan. East Mediterr Health J.

[9] Haddad LG, Shotar A, Younger JB, Alzyoud S, Bouhaidar CM (2011). Screening for domestic violence in Jordan: validation of an Arabic version of a domestic violence against women questionnaire. Int J Womens Health.

[10] The Euro-Mediterranean Human Rights Monitor (2020). Women in Jordan Continuing Violence and Absent Protection.

[11] Kishor (2005). Domestic violence measurement in the demographic and health surveys: The history and the challenges. Measure.

[12] Straus MA (2017). Measuring intrafamily conflict and violence: The conflict tactics (CT) scales. Physical Violence in American Families.

[13] Heise LL, Pitanguy J, Germain A (1994). Violence against women: the hidden health burden. World Bank Discuss Pap.

[14] Rutstein SO, Johnson K (2004). The DHS Wealth Index. DHS Comparative Reports No 6.

[15] Martel P, Mbofana F, Cousens S (2021). The polychoric dual-component wealth index as an alternative to the DHS index: Addressing the urban bias. J Glob Health.

[16] Poirier MJP, Grépin KA, Grignon M (2020). Approaches and alternatives to the wealth index to measure socioeconomic status using survey data: A critical interpretive synthesis. Soc Indic Res.

[17] WHO (2001). Putting women first: Ethical and Safety Recommendations for Research on Domestic Violence Against Women. https://apps.who.int/iris/handle/10665/65893.

[18] von Elm E, Altman DG, Egger M, Pocock SJ (2007). ; STROBE Initiative. The Strengthening the Reporting of Observational Studies in Epidemiology (STROBE) statement: guidelines for reporting observational studies. Ann Intern Med.

[19] Elghossain T, Bott S, Akik C, Obermeyer CM (2019). Prevalence of intimate partner violence against women in the Arab world: a systematic review. BMC Int Health Hum Rights.

[20] Pierotti RS (2013). Increasing rejection of intimate partner violence: Evidence of global cultural diffusion. Am Sociol Rev.

[21] Yount KM, Halim N, Schuler SR, Head S (2013). A survey experiment of women's attitudes about intimate partner violence against women in rural Bangladesh. Demography.

[22] Krause KH, Gordon-Roberts R, VanderEnde K, Schuler SR, Yount KM (2016). Why Do Women Justify Violence Against Wives More Often Than Do Men in Vietnam?. J Interpers Violence.

[23] Sardinha L, Nájera Catalán HE (2018). Attitudes towards domestic violence in 49 low-and middle-income countries: A gendered analysis of prevalence and country-level correlates. PLoS One.

[24] Andersson N, Ho-Foster A, Mitchell S, Scheepers E, Goldstein S (2007). Risk factors for domestic physical violence: national cross-sectional household surveys in eight southern African countries. BMC Womens Health.

[25] Gautam S, Jeong HS (2019). Intimate Partner Violence in Relation to Husband Characteristics and Women Empowerment: Evidence from Nepal. Int J Environ Res Public Health.

[26] Lasong J, Zhang Y, Muyayalo KP, Njiri OA (2020). Domestic violence among married women of reproductive age in Zimbabwe: a cross sectional study. BMC Public Health.

[27] Peltzer K, Pengpid S (2014). Female genital mutilation and intimate partner violence in the Ivory Coast. BMC Womens Health.

[28] Benebo FO, Schumann B, Vaezghasemi M (2018). Intimate partner violence against women in Nigeria: a multilevel study investigating the effect of women's status and community norms. BMC Womens Health.

[29] Chernet AG, Cherie KT (2020). Prevalence of intimate partner violence against women and associated factors in Ethiopia. BMC Womens Health.

[30] Beni Yonis O, Khader Y, Al-Mistarehi AH, Abu Khudair S, Dawoud M (2021). Behavioural and emotional symptoms among schoolchildren: a comparison between Jordanians and Syrian refugees. East Mediterr Health J.

[31] Gibbs A, Jewkes R, Willan S, Washington L (2018). Associations between poverty, mental health and substance use, gender power, and intimate partner violence amongst young (18-30) women and men in urban informal settlements in South Africa: A cross-sectional study and structural equation model. PLoS One.

[32] Peterman A, Pereira A, Bleck J, Palermo TM, Yount KM (2017). Women's Individual Asset Ownership and Experience of Intimate Partner Violence: Evidence From 28 International Surveys. Am J Public Health.

[33] Coll CVN, Ewerling F, García-Moreno C, Hellwig F, Barros AJD (2020). Intimate partner violence in 46 low-income and middle-income countries: an appraisal of the most vulnerable groups of women using national health surveys. BMJ Glob Health.

[34] Kargar Jahromi M, Jamali S, Rahmanian Koshkaki A, Javadpour S (2015). Prevalence and Risk Factors of Domestic Violence Against Women by Their Husbands in Iran. Glob J Health Sci.

[35] Sapkota D, Bhattarai S, Baral D, Pokharel PK (2016). Domestic violence and its associated factors among married women of a village development committee of rural Nepal. BMC Res Notes.

